# Fibroblast growth factor 18 stimulates chondrocyte proliferation by modulating FOXN2 to mitigate post-traumatic osteoarthritis in a mouse model

**DOI:** 10.3389/fbioe.2025.1615124

**Published:** 2025-11-13

**Authors:** Zhusong Huang, Wenhan Zhao, Jinfu Lan, Yu Lin, Xi Gao

**Affiliations:** Department of Orthopedic Surgery, Fuzhou Second General Hospital, Fuzhou, Fujian, China

**Keywords:** fibroblast growth factor 18 (FGF18), forkhead box N2 (FOXN2), bone morphogenetic protein 2 (BMP2), C-telopeptide of type II collagen (CTX-II), a disintegrin and metalloproteinase with thrombospondin motifs 5 (ADAMTS-5), post-traumatic osteoarthritis (PTOA), destabilized medial meniscus (DMM) model

## Abstract

**Introduction:**

FGF18 is linked to osteoarthritis (OA) progression, but its relationship with FOXN2 and its roles in post-traumatic osteoarthritis (PTOA) remain unclear.

**Methods:**

We conducted comparative screening between PTOA and normal controls to assess FGF18 expression in articular cartilage. Functional studies examined FGF18 overexpression effects on chondrocyte proliferation and cartilage degradation. Intra-articular FGF18 delivery was performed in destabilized medial meniscus (DMM)-induced PTOA mice models.

**Results:**

FGF18 expression was significantly downregulated in articular cartilage of PTOA patients compared to normal cases. FGF18 overexpression enhanced chondrocyte proliferation through BMP2 upregulation and attenuated cartilage degradation by suppressing CTX-II and catabolic factors (MMP13 and ADAMTS-5), while substantially promoting aggrecan synthesis. Intra-articular FGF18 delivery in DMM mice significantly reduced cartilage erosion and markedly decreased synovial thickening compared to saline-treated controls, with improved cartilage matrix integrity. FOXN2 expression was significantly upregulated in FGF18-knockout chondrocytes but restored upon FGF18 overexpression.

**Discussion:**

These findings highlight that FGF18 mitigates PTOA progression by targeting FOXN2, promoting robust aggrecan synthesis, and substantially suppressing cartilage-degrading enzymes. Our study delineates a novel therapeutic axis for PTOA, emphasizing the distinct molecular mechanisms underlying trauma-driven cartilage pathology.

## Introduction

Post-traumatic osteoarthritis (PTOA) is a prevalent subtype of OA with significant epidemiological burden. The prevalence of PTOA accounts for approximately 12% of all symptomatic osteoarthritis cases, representing roughly 5.6 million cases of lower extremity OA in the United States (Brown et al., 2006). The incidence of knee injuries, a primary risk factor for PTOA development, ranges from 2.29 to 12 cases per 1,000 inhabitants per year, with the highest incidence occurring in individuals aged 15–24 years, often resulting from joint injuries such as ligament tears or fractures (Gage et al., 2012). Following intra-articular fractures, the prevalence of PTOA development reaches 23%–36% of patients, highlighting the significant risk associated with traumatic joint injuries (Anderson et al., 2011). Unlike primary OA, which is linked to aging, PTOA arises from acute mechanical trauma or chronic overload, leading to accelerated cartilage degradation and joint dysfunction (Martin and Buckwalter, 2006). While PTOA shares end-stage joint destruction with idiopathic OA, its pathogenesis involves distinct mechanisms, including oxidative stress-induced chondrocyte damage and altered joint biomechanics post-injury (Robbins et al., 2019a; Robbins et al., 2019b).

Current therapies for PTOA primarily focus on symptom management, such as pain relief and surgical interventions. However, treatments targeting the underlying pathology—such as cartilage restoration or prevention of post-traumatic cartilage degeneration—remain limited (Stiebel et al., 2014; Alves et al., 2020). Moreover, current biologics such as hyaluronic acid and platelet-rich plasma may have limited efficacy in addressing PTOA-specific pathology, particularly the acute post-traumatic inflammatory response and rapid cartilage degradation that distinguish PTOA from age-related osteoarthritis. Emerging approaches include biologics to modulate oxidative stress (Martin and Buckwalter, 2006), joint-unloading implants (Stiebel et al., 2014), and strategies addressing nociceptive mechanisms (Robbins et al., 2019b). Notably, biomechanical studies reveal differences in cartilage thickness patterns6, muscle activation (Robbins et al., 2019a), and alignment-related joint loading between PTOA and non-traumatic OA, suggesting the need for subtype-specific therapeutic strategies (Robbins et al., 2019a; Robbins et al., 2019b). Despite advances, significant gaps persist in understanding how to mitigate cartilage degradation or restore joint homeostasis after trauma, highlighting the urgency for mechanistically targeted therapies in PTOA management (Stiebel et al., 2014).

Fibroblast growth factors (FGFs) are an integral part of the growth factor family and participate in processes such as cellular and developmental processes. FGFs have been implicated in the onset of diseases and consequently their treatment (Jan et al., 2023). Various FGF members have been extensively studied including FGF18, and it has been established to be involved in cartilage repair (Yun et al., 2010). Fibroblast growth factor 18 is essential for proper cell proliferation and differentiation during osteogenesis and chondrogenesis. FGF18 is a protein that increases chondrocyte proliferation and is also an osteogenic stimulant (Hendesi et al., 2022). A study by Yao Xudong et al. found that FGF18 attenuates cartilage, increases collagen II deposition, and suppresses matrix metallopeptidase 13 (MMP13) expression in rats with post-traumatic OA (Lv et al., 2024). Furthermore, recombinant human FGF18 has been under development by Merk as sprifermin, a drug with the potential to act as a disease-modifying compound for the treatment of osteoarthritis. This drug has been observed to induce cartilage growth and maintenance in patients with OA over a period span of 3.5 years (Eckstein et al., 2021).

The Foxhead box (Fox) family, an evolutionarily conserved family of transcription factors expressing the Foxhead motif, is critical to human health and disease (Laissue, 2019). The FOXN subfamily members of the forkhead transcription factors have only been discovered. The FOXN subfamily is composed of six members including FOXN1, FOXN2, FOXN3, FOXN4, FOXN5, and FOXN6 (Rho and McClay, 2011). Novel research is being conducted on the regulatory function of the Fox family in relation to bone metabolism and orthopedic disorders. Fox factors are involved in the development of osteoarthritis and rheumatoid arthritis (Xu et al., 2021). Research has determined that FOXN2 participates in roles directing hypertrophic chondrocyte differentiation (Li et al., 2016) and it has been incorporated into the chondrocyte core gene regulatory network (Gomez-Picos et al., 2022). Genetic research studies have indicated that the Fox gene family participates in bone metabolism and numerous factors can regulate their expression. Their dysregulation can lead to a number of bone diseases (Huang et al., 2020). Studies have identified regulatory roles between FGF18 and fox family members such as FOXF2 in cleft palate development (Xu et al., 2016), however, there is little to no research that has gone into establishing FOXN2 in the development of PTOA or its regulation by FGF18 in PTOA, and how their interaction may lead to or prevent the onset of osteoarthritis.

The regulation of FOXN2 by FGF18 aids in maintaining the homeostatic balance of factors that play crucial roles in PTOA. These important factors include collagenase matrix metalloproteinase 13 (MMP-13), disintegrin and metalloproteinase with thrombospondin motifs (ADAMTS)-5, collagen, and aggrecan. The basic structural elements of cartilage are collagen and aggrecan, and the deterioration of these materials is correlated with the advancement of PTOA. MMP-13 and ADAMTS-5 are collagenase and aggrecanase, respectively (Yang et al., 2017), which means they are responsible for the degradation of type II collagen and aggrecan.

Based on the gaps in current understanding and the promising role of FGF18 in cartilage homeostasis, the aims of this study were to: (1) determine whether FGF18 can mitigate PTOA cartilage degradation through regulation of FOXN2, and (2) investigate the molecular mechanisms by which FGF18 influences chondrocyte function and cartilage matrix homeostasis in PTOA progression. We hypothesized that FGF18 promotes chondrocyte proliferation and suppresses cartilage-degrading enzymes via upregulation of FOXN2, thereby attenuating PTOA progression. To test this hypothesis, we employed both *in vitro* human chondrocyte studies and *in vivo* destabilized medial meniscus (DMM) mouse models to elucidate the FGF18-FOXN2 regulatory axis and its therapeutic potential in PTOA management.

## Materials and methods

### Study design

This study employed both *in vitro* and *in vivo* experimental designs, with *in vitro* experiments using human chondrocytes for FGF18 overexpression and knockdown, and *in vivo* experiments using a DMM mouse model to evaluate FGF18 therapeutic effects. The independent variable was FGF18 expression level (overexpression, knockdown, or control), and dependent variables included chondrocyte proliferation, apoptosis, FOXN2 expression, and PTOA-related markers (MMP13, ADAMTS-5, CTX-II, aggrecan).

### Ethics statement

The usage of human samples in this study has been approved by the Fuzhou Second General Hospital (Approval No. 2022140), and informed written consent was obtained from all participants prior to surgery for both specimen collection and research participation. All animal experimental procedures were approved by the Experimental Animal Ethics Committee of Fujian Medical University (Approval No. IACUC FJMU 2024-0248).

### Target gene selection for PTOA research

Based on comprehensive literature review of factors involved in cartilage development, repair and osteoarthritis pathogenesis, FGF18 was identified as a promising candidate gene for investigation in PTOA. Previous studies have established FGF18 as an important regulator of chondrocyte proliferation and cartilage formation (Davidson et al., 2005; Ellsworth et al., 2002), and as a therapeutic target with recombinant FGF18 (sprifermin) in clinical trials for primary OA (Hochberg et al., 2019; Eckstein et al., 2021). However, its specific roles and regulatory mechanisms in post-traumatic osteoarthritis remained poorly understood, warranting further investigation (Buniello et al., 2019; MacArthur et al., 2017; Welter et al., 2014).

### Collection of human samples

Articular cartilage tissue was obtained from knee biopsies of patients undergoing total knee arthroplasty (PTOA group, n = 10), and non-OA specimens were sourced from individuals undergoing surgery due to acute traumatic injuries, including fractures or ligament tears, with no prior history of osteoarthritis, as normal controls (NC group, n = 8). Once obtained, the cartilage was immediately placed in liquid nitrogen. The cartilage tissues were processed for further histological examinations.

### Animals

Male C57BL/6 mice weighing 200 g (8 weeks old) were purchased from the Experimental Animal Centre of Tongji Medical College (Wuhan, China), a nationally certified SPF facility that provides genetically standardized laboratory animals. After transportation, mice underwent a 7-day acclimatization period at the animal facility of Fujian Medical University before experimental procedures. All animal experiments were conducted at the accredited animal facility of Fujian Medical University under strict adherence to institutional and national ethical guidelines.

### OA mice model construction

For the OA experiment model, the mice were placed under anesthesia using sodium pentobarbital (100 mg/kg) administered intraperitoneally by injection. Then the mice were traversed by the medial collateral ligament and destabilized by the medial meniscus (DMM) in the right knee. Then the mice were injected with replication-deficient lentivirus (Lenti)-FGF18 and saline via intra-articular administration 3 days, 1 week, 2 weeks, and 3 weeks after the DMM surgery. The mice were sacrificed 8 weeks after the operation by the cervical dislocation method, and the knee joints were harvested.

### Cell culture and expansion

Articular cartilage from OA patients and non-OA individuals as well as from the knees of mice after the construction of the OA model, were isolated as previously described (Hendriks et al., 2006). In short, cartilage was separated from the underlying bone and connective tissue and digested in collagenase type II (0.15% Worthington) in DMEM supplemented with 100 mg/mL of streptomycin and 100 U/mL of penicillin for 20–22 h. Chondrocyte proliferation medium (DMEM supplemented with 10% FBS, 1 × nonessential amino acids, 0.2 mM Ascorbic acid 2-phosphate, 0.4 mM proline, 100 U/mL penicillin, and 100 μg/mL streptomycin) was then used to cultivate the primary chondrocytes.

### Cell transfection

The FGF18 sequence was synthesized and subcloned into the pcDNA3.1 (Invitrogen, Life Technologies, Carlsbad, CA, United States) vector. Overexpression of FGF18 was achieved via pcDNA3.1-FGF18 transfection, with an empty pcDNA3.1 vector used as a control (Invitrogen). Negative control siRNA or siRNA against FGF18 (Sigma-Aldrich, St. Louis, MO, United States) was transfected into chondrocytes at a concentration of 50 µM using Lipofectamine 3000 Transfection Reagent (Invitrogen). Transfection was performed for 6 h at 37 °C, followed by medium replacement and a 48-h recovery period before subsequent functional analyses.

### Histological and immunohistochemical analysis of PTOA

Human cartilage OA tissues (OA) and non-OA cartilage samples (NC) were fixed in 4% neutral-buffered paraformaldehyde (PFA: Salarbio, Beijing, China), embedded in paraffin and sliced with a rotary microtome into 5 µM slices. The cartilage sections were then stained with hematoxylin and eosin, Safranin O 0.25% v/v (Salarbio) for 5 min with Fast Green 0.01% v/v (f7258: Sigma-Aldrich, St Louis, MO, United States) as a counterstain.

The knee joints of DMM-induced OA mice were fixed in 10% formaldehyde at 4 °C for approximately 24 h. The cartilage tissues were then decalcified in 0.5 M ethylenediaminetetraacetic acid in PBS (pH 7.4) for 2 weeks. Then the specimens were embedded in paraffin, sliced into 5 µM sections, and stained with hematoxylin and eosin, Safranin O, and Fast Green.

For immunohistochemical staining, paraffin-embedded human cartilage sections from OA patients and NC individuals were treated with 3% hydrogen peroxide to block the activity of endogenous peroxidase and then incubated in 10% goat serum in PBS. Thereafter, the sections were incubated with rabbit anti-FGF18 (Invitrogen, Waltham, MA, #PA5-106562) diluted 1:100 in 10% goat serum in PBS overnight, then thoroughly washed and incubated with goat anti-rabbit immunoglobulin G (Vector Laboratories, Burlingame, CA, #BA-1000) diluted in 1:100 PBS. ABC kit (Vector Laboratories, #PK-4000) was used for detection followed by the DAB kit (Vector Laboratories, #SK-4100) according to the manufacturer’s protocol. Quantification of anti-FGF18 positive staining was performed usi ng ImageJ.

### Immunofluorescence assay

For immunofluorescence staining, chondrocytes from patients that were transfected with indicated plasmids, and chondrocytes from DMM-induced OA mice transfected with Lenti-FGF18 and saline (control) were analyzed using immunofluorescence microscopy for the expression of CTX-II (both human and mice chondrocytes), BMP-2 (human chondrocytes only), Aggrecan (mice chondrocytes only), MMP13 (mice chondrocytes only), and ADAMTS-5 (mice chondrocytes only). After washing three times with PBS, cells were fixed with 4% formaldehyde for 10 min at room temperature, followed by blocking with 2.5% BSA for 1 h at room temperature. Cells were subsequently incubated with anti-CTX-II (1:500; ab3092; Abcam; validated for human and mouse), anti-BMP-2 (1:500; ab6285; Abcam; human-specific), anti-Aggrecan (1:500; ab36861; Abcam; mouse-specific), anti-MMP13 (1:500; ab39012; Abcam; mouse-specific), anti-ADAMTS-5 (1:500; ab41037; Abcam; mouse-specific) overnight at 4 °C in DPBS containing 2.5% BSA and 0.1% Tween 20. Samples were then washed three times with Dulbecco’s Phosphate-Buffered Saline (DPBS), followed by probing with Fluorescein isothiocyanate (FITC) or Tetramethylrhodamine (TRITC) conjugate secondary antibody (1:1000; Thermo Fisher Scientific) at room temperature for 1 h. Thereafter, cells were counterstained with DAPI and analyzed using fluorescence microscope.

### qRT-PCR assay

Total RNA was extracted from chondrocytes using RNA isolater Tatol RNA Extraction Reagent (Vazyme, China). Purified RNA was reverse-transcribed into cDNA with HiScript^®^ II 1st Strand cDNA Synthesis Kit (Vazyme, China) according to the manufacturer’s protocol. Real-time qRT-PCR was performed using the Applied Biosystems StepOnePlus Real-Time PCR System (Foster City, CA, United States) with SYBR Green PCR Master Mix (Toyobo, Japan). The primers used for qRT-PCR were as follows: FGF18 forward: 5′-CAA​GGA​GTG​CGT​GTT​CAT​TGA​GA-3′, reverse: 5′-CGG​GAT​CGC​TTA​GTA​ACT​GTG​GT-3'; GAPDH forward: 5′-GGC​AAG​TTC​AAC​GGC​ACA​G-3′, reverse: 5′-GCC​AGT​AGA​CTC​CAC​GA-3'. To quantify the relative expression levels of mRNA, the values were normalized against the endogenous reference GAPDH. The 2^(-ΔΔCT) method was used for data analysis. Additionally, qRT-PCR was employed to validate RNA-sequencing results, with independent training (n = 6) and validation sets (n = 6) used to confirm the expression of key differentially expressed genes, such as FOXN2, across datasets.

### RNA-sequencing analysis

Total RNA was extracted with TRIzol (Invitrogen) from OA and NC chondrocytes. The purity and quantity of total RNA were measured by Nanodrop. The integrity of RNA was evaluated using the RNA Nano6000 Assay Kit on the Bioanalyzer 2100 system (Agilent Technologies, CA, United States). A total of 1 µg RNA per sample was used as an input for further analysis. Strand-specific RNA-sequencing libraries were generated using the NEBNext Ultra II RNA Library Prep Kit (Illumina, United States). Library quality was evaluated on the Agilent Bioanalyzer 2100 system (Agilent Technologies, CA, United States). Final libraries were sequenced on the Illumina Novaseq 6000 platform by 150 bp paired-end reads. The expression matrix was normalized and differentially expressed genes (DEG) were searched using the R package DeSeq2 (52) (thresholds used: adjusted p-value <0.01 and log2(Fold-Change) > 0.58). Supplementary R packages compiled with R version 4.1.1 were used to generate data visualization: ComplexHeatmap (2.8.0).

### Western blotting

For total protein extraction, primary chondrocytes of OA patients (OA) and non-OA individuals (NC) were lysed in RIPA buffer (Fudebio, Hangzhou, China) containing protease inhibitor cocktails and phosphatase inhibitors (Fudebio, Hangzhou, China). A bicinchoninic acid protein assay kit (Thermo Fisher Scientific) measured the protein quantities. Equivalent amounts of protein were separated by SDS-PAGE and transferred onto polyvinylidene fluoride (PVDF) membranes (Merck KGaA). After incubation with anti-FGF18 antibody (1:1000, Proteintech) or anti-GADPH (1:1000, Cell Signaling Technology, United States). After that, the membranes were treated with horseradish peroxidase (HRP)-conjugated secondary antibodies (FDM007 and FDR007, Fudebio, Hangzhou, China). Following washing, an enhanced chemiluminescence kit (FD8030, Fudebio, Hangzhou, China) was utilized to identify the band signals. Furthermore, primary chondrocytes after being transfected with pcDNA3.1, pcDNA3.1-FGF18, control siRNA, and FGF18 siRNA were analyzed for the expression of FOXN2, Col II, MMP13 and ADAMTS-5 using primary antibodies against collagen type II (ab3778; Abcam, Cambridge, United Kingdom), MMP13 (sc-515284; Santa Cruz Biotechnology, Dallas, TX, United States), ADAMTS-5 (MAB2198; R&D Systems, Minneapolis, MN, United States), and FOXN2 (#12941, Cell Signaling Technology, Danvers, MA, United States) following instructions as discussed above.

### Flow cytometry analysis

Human articular chondrocytes transfected with pcDNA3.1, pcDNA3.1-FGF18, control siRNA, and FGF18 siRNA were collected using trypsin without EDTA (Thermo Fisher Scientific, Waltham, MA, United States) and washed with PBS twice. The cells were then stained using Annexin V-fluorescein isothiocyanate/propidium iodide (FITC/PI) Apoptosis kit (BD Biosciences, Franklin Lakes, NJ, United States) according to the manufacturer’s protocol. BD FACS flow cytometer (BD Biosciences) was used to identify cellular apoptosis.

For determining transfection efficiency, trypsin was used to remove chondrocytes from culture vessels. Cells were recovered in PBS supplemented with 2% FBS after 5 min. Then green fluorescent protein (GFP), expressed by the cells was analyzed in chondrocytes utilizing Beckman Coulter’s Kaluza analytical software and flow cytometry (EPICS XL FACS).

### Cell proliferation assay

EDU (5-ethynyl-20-Deoxyuridine) assay was used to evaluate chondrocyte proliferation abilities after they were transfected with pcDNA3.1, pcDNA3.1-FGF18, control siRNA, and FGF18 siRNA. EDU staining was performed according to the manufacturer’s protocol using EDU detecting kits (Ribobio, Guangzhou, China). Briefly, EDU was added at a final concentration of 10 µM and the cells were incubated for 2 h at 37 °C. After labeling, the cells were fixed with 4% PFA for 15 min and permeabilized with 0.2% Triton X-100 for 10 min. The cells were then stained in the dark with click reaction solution for 30 min before being counterstained with Hoechst 33342. Using fluorescence microscopy (Olympus, Tokyo, Japan). EDU-positive cells were counted and the percentage was reported.

### Statistical analysis

Data are presented as means ± SD unless otherwise specified. For comparisons between two groups, two-tailed Student's t-tests were used. For multiple group comparisons, one-way analysis of variance (ANOVA) with Tukey’s *post hoc* test was applied. For RNA-sequencing data, normalization and differential expression analysis were conducted using the DESeq2 package with a threshold of adjusted P < 0.05 and |log2FoldChange|>1. Sample sizes were determined based on power calculations (α = 0.05, β = 0.2) from preliminary experiments. All statistical analyses were conducted by investigators blinded to group allocation using GraphPad Prism software (version 9.0). P values less than 0.05 were considered statistically significant.

## Results

### FGF18 expression patterns in PTOA and NC articular cartilage

**FIGURE 1:**
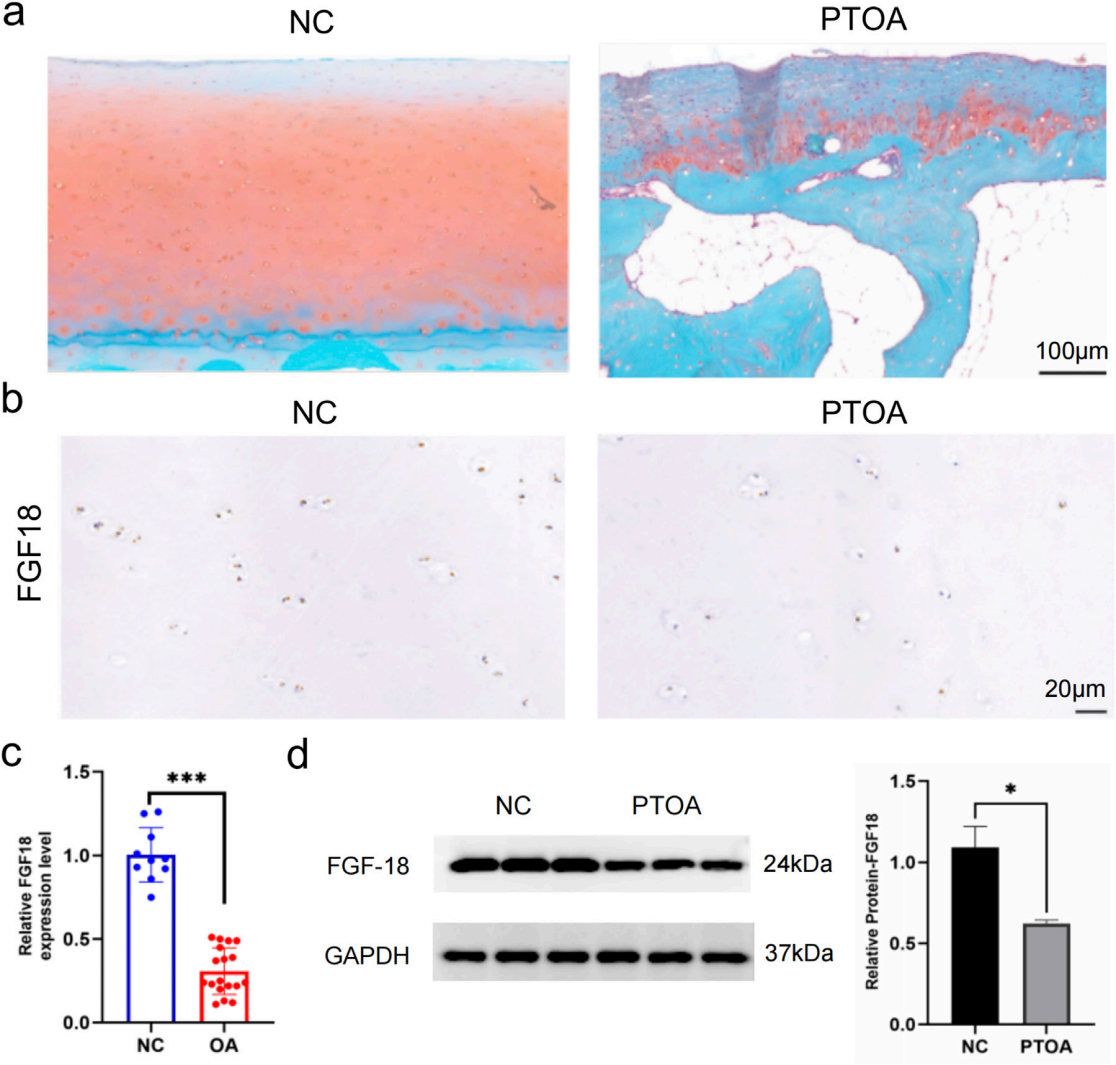
The expression pattern of FGF18 in PTOA and normal control cartilage. **(a)** Representative gross appearance of cartilage of PTOA patients and normal control patients. **(b)** The analysis of FGF18 expression in the cartilage of PTOA and normal control patients using immunohistochemical staining. **(c)** qRT-PCR of the relative expression of FGF18 in the cartilage PTOA and normal control patients. **(d)** Western blot analysis illustrating the expression of FGF18 in the cartilage of PTOA and normal control patients.

The expression of FGF18 in the cartilage from PTOA and NC trauma patients was explored to identify its role in osteoarthritis development or progression. First, using histological analysis, the morphology of the cartilage was inspected. The histological results from PTOA patients showed that the cartilage was damaged as opposed to the cartilage from NC patients (Figure 1a). Then, using immunochemical analysis, the expression levels of FGF18 were observed (Figure 1b) and the expression patterns of FGF18 were significantly downregulated in the cartilage of patients with PTOA. The results were quantified using real-time RT-PCR and Western blot analysis (Figures 1c,d). These results illustrate that FGF18 may have a functional role in PTOA development or progression.

### FGF18 regulates chondrocyte proliferation to prevent cartilage degradation

**FIGURE 2:**
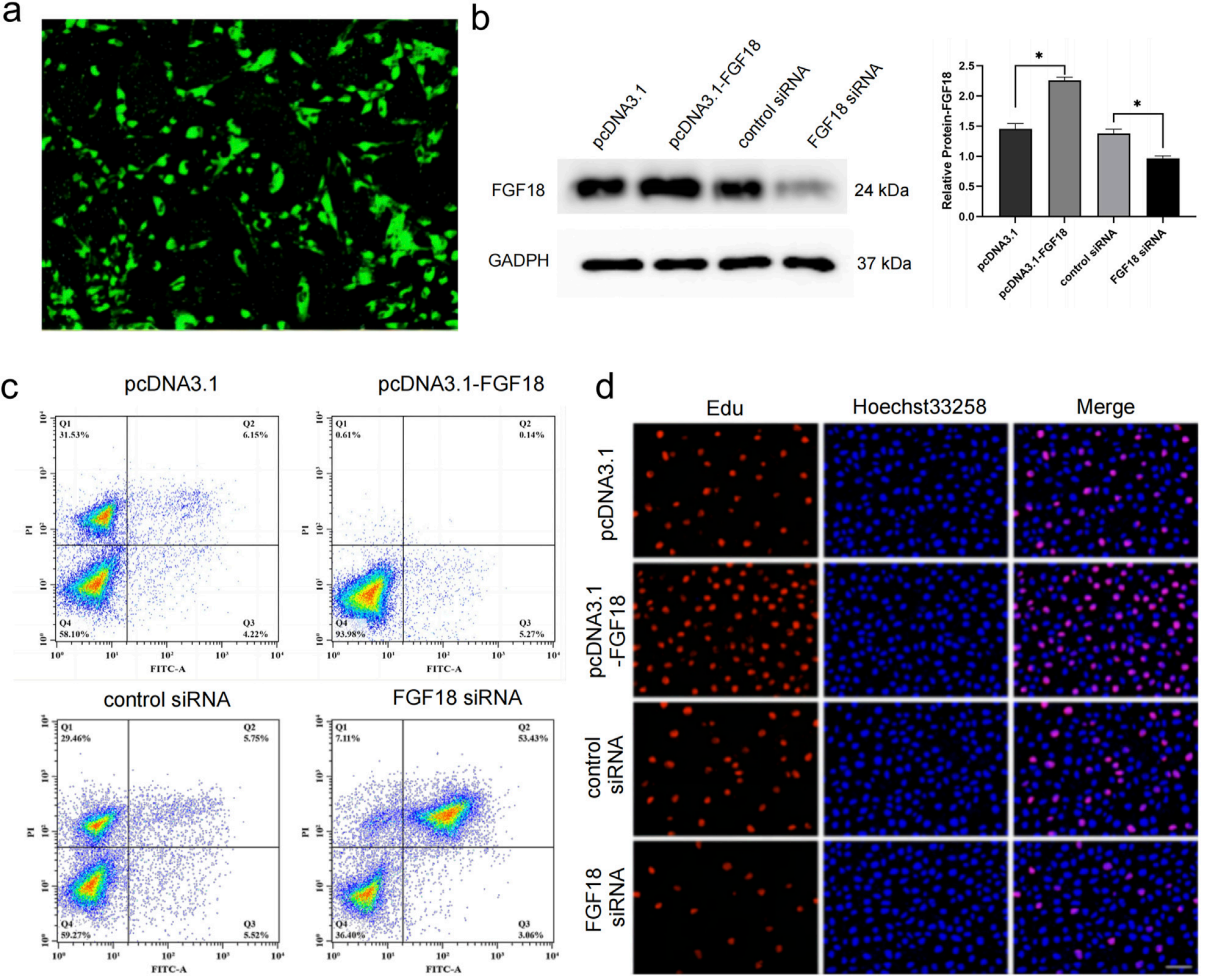
FGF18 regulates the proliferation of chondrocytes. **(a)** Analysis of transfection efficiency of pcDNA3.1-FGF18 in human chondrocytes using green fluorescent protein (GFP). **(b)** Immunoblot analysis illustrating the expression levels of FGF18 in human chondrocytes transfected with pcDNA3.1-FGF18, FGF18 siRNA, or their corresponding controls. n = 6 independent biological replicates per group. **(c)** Analysis of human chondrocyte apoptosis transfected with indicated plasmids assayed by flow cytometry. n = 6 independent biological replicates per group. **(d)** Analysis of human chondrocyte proliferation transfected with indicated plasmids assayed by EdU assay. n = 6 independent biological replicates per group.

FGF18 has been implicated in chondrogenesis and osteogenesis thereby stimulating the formation of cartilage (Jan et al., 2023; Wu et al., 2022). This action implies that FGF18 may prevent or improve PTOA. Thus low expression of FGF18 could potentially be associated with poor cartilage regeneration. To determine this, primary chondrocytes from PTOA patients were transfected with the overexpression of FGF18 or FGF18 knockdown, and their controls. The transfection efficiency was analyzed to validate the success of the transfection (Figure 2a). The expression of FGF18 was elevated when FGF18 was overexpressed but downregulated when FGF18 was silenced (Figure 2b). The level of cellular apoptosis of chondrocytes after transfection with FGF18 siRNA was elevated when compared to its control, while the level of apoptosis was decreased when pcDNA3.1-FGF18 was transfected compared to its control (Figure 2c). Furthermore, the EDU assay demonstrated that chondrocyte proliferation was significantly higher after the transfection of FGF18 overexpression into the chondrocytes but lower after the transfection of FGF18 knockdown (Figure 2d). This implicates FGF18 in governing the proliferation of chondrocytes, which may prevent matrix breakdown.

### The knockdown of FGF18 upregulates the expression of FOXN2 and affects PTOA-related factors

**FIGURE 3:**
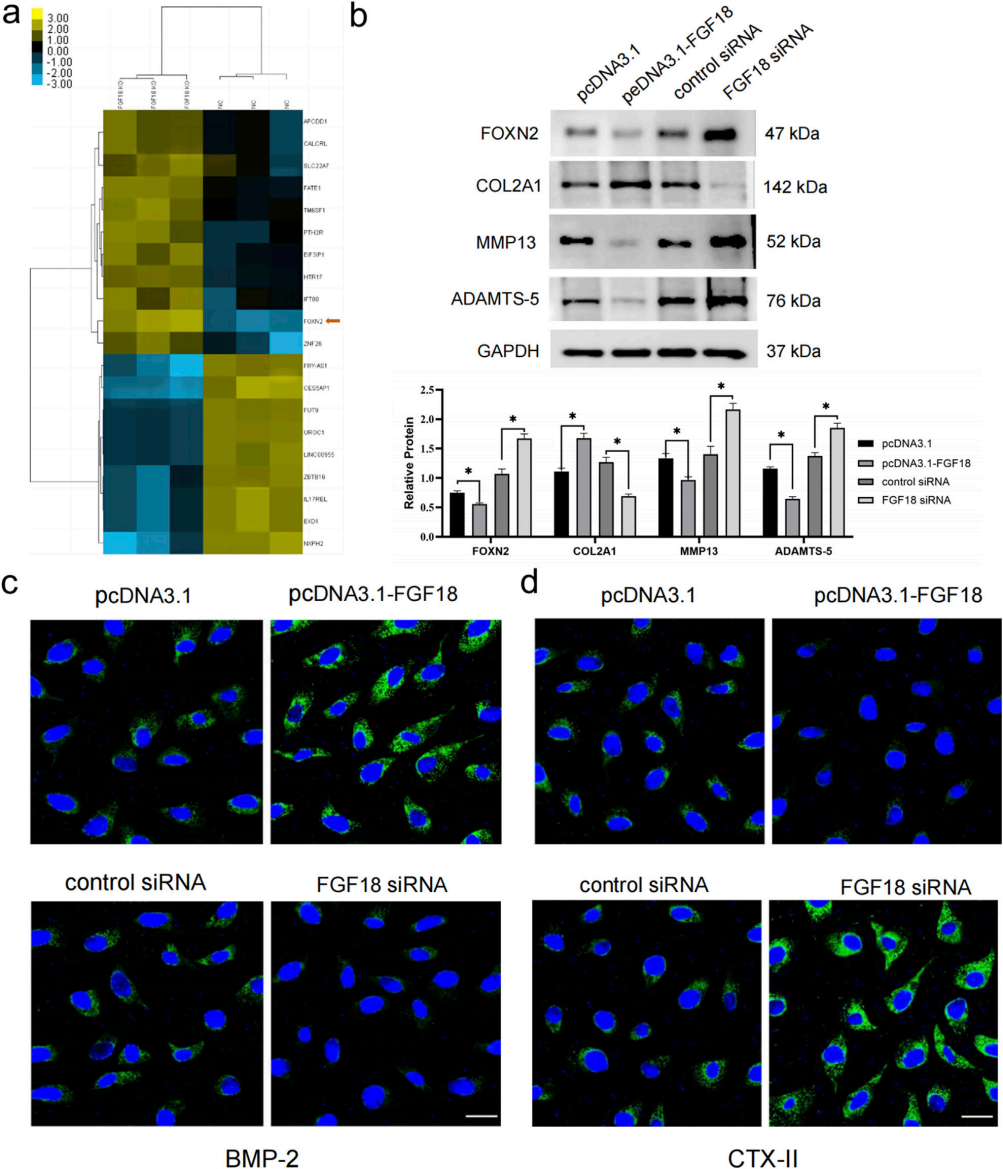
FGF18 regulates the expression level of FOXN2 and PTOA-associated factors. **(a)** Heat map demonstrating differentially expressed genes in FGF18-knockout chondrocytes compared to normal controls. Statistical significance was determined using DESeq2 with adjusted P < 0.05 and |log2 Fold Change|>1. **(b)** Western blot analysis illustrating the expression levels of FGF18 in human chondrocytes transfected with indicated plasmids. n = 6 independent biological replicates per group. FOXN2 was the target gene of FGF18. Statistical significance: *P < 0.05, **P < 0.01, ***P < 0.001 vs. corresponding controls. **(c,d)** Analysis of BMP-2 and CTX-II expression in human chondrocytes transfected with indicated plasmids using immunofluorescence assay.

To comprehensively identify the mechanism of FGF18 in the development or progression of PTOA, we performed RNA-seq analysis in chondrocytes from PTOA patients. It was found that FOXN2 was among the differentially expressed genes (Figure 3a). The RNA-seq analysis was conducted on *in vitro* human chondrocytes from normal controls (NC, n = 3 biological replicates) and FGF18 knockout (FGF18 KO, n = 3 biological replicates), revealing 19 differentially expressed mRNAs between FGF18 knockout cells and normal controls, as summarized in Table 1 for both the training and validation sets. Among these, FOXN2 showed robust upregulation in FGF18 KO cells (log2FC ≈ 3.60, p = 0.001 in RNA-seq; training set: fold change 2.5, p = 0.006; validation set: 2.55, p = 0.004), ranking among the most significantly altered genes. FOXN2 was prioritized for further validation over other DEGs due to its significant fold-change, statistical significance, consistency across training and validation datasets, and established biological relevance to chondrocyte differentiation and hypertrophy. Then we determined that FOXN2 was upregulated when FGF18 was knocked down, this suggests that FOXN2 is a molecular target of FGF18 in the regulatory process of PTOA. Factors associated with PTOA such as MMP13 and ADAMTS-5 were also observed to be upregulated (Figure 3b). Immunofluorescence staining in Figure 3c,d specifically highlights the expression of BMP-2 (Bone Morphogenetic Protein-2, a key pro-anabolic factor that promotes cartilage matrix synthesis and repair by stimulating collagen production and chondrocyte proliferation) and CTX-II (a biomarker of type II collagen degradation) in chondrocytes under FGF18 knockdown conditions. BMP-2 exhibited reduced fluorescence intensity in FGF18-silenced chondrocytes, while CTX-II was markedly upregulated. The diminished BMP-2 signal suggests impaired cartilage repair capacity, whereas elevated CTX-II reflects enhanced collagen breakdown. These results indicate that FGF18 knockdown disrupts the balance between anabolism (via BMP-2 suppression) and catabolism (via MMP13, ADAMTS-5 and CTX-II upregulation), exacerbating cartilage degeneration in PTOA. From these results, we gathered that FGF18 regulates FOXN2 and prevents or counteracts the pathological onset of PTOA. We surmise that the downregulation of FGF18 in PTOA results in the upregulation of FOXN2 together with MMP13, ADAMTS-5 and BMP-2, factors associated with PTOA.

**TABLE 1:** Differentially expressed mRNAs in FGF18 knockout cells compared with normal controls in both the training set and the validation set.

mRNA	Training set		Validation set	
	Fold change P value		Fold change P value	
Upregulated				
APCDD1	1.8	0.25	-	-
PTH2R	1.9	0.16	-	-
FATE1	1.7	0.22	-	-
TM6SF1	1.6	0.16	-	-
FOXN2	**2.5**	0.006 **	**2.55**	0.004 **
SLC22A7	1.5	0.15	-	-
CALCRL	1.7	0.2	-	-
ZNF26	2	0.17	-	-
IFT80	1.8	0.13	-	-
EIF3IP1	**2.1**	0.006 **	**2.15**	**0.18**
HTR1F	2.3	0.13	-	-
Downregulated				
FUT9	**0.45**	0.008 **	**0.47**	**0.12**
FRY-AS1	**0.35**	0.007 **	**0.37**	**0.15**
CES5AP1	0.4	0.21	-	-
LINC00955	0.5	0.2	-	-
ZBTB16	0.45	0.18	-	-
UROC1	0.55	0.22	-	-
IL17REL	0.4	0.17	-	-
NXPH2	0.35	0.15	-	-
EXD1	0.45	0.2	-	-

**P < 0.01, ***P < 0.001.

### The overexpression of FGF18 attenuates PTOA progression

**FIGURE 4:**
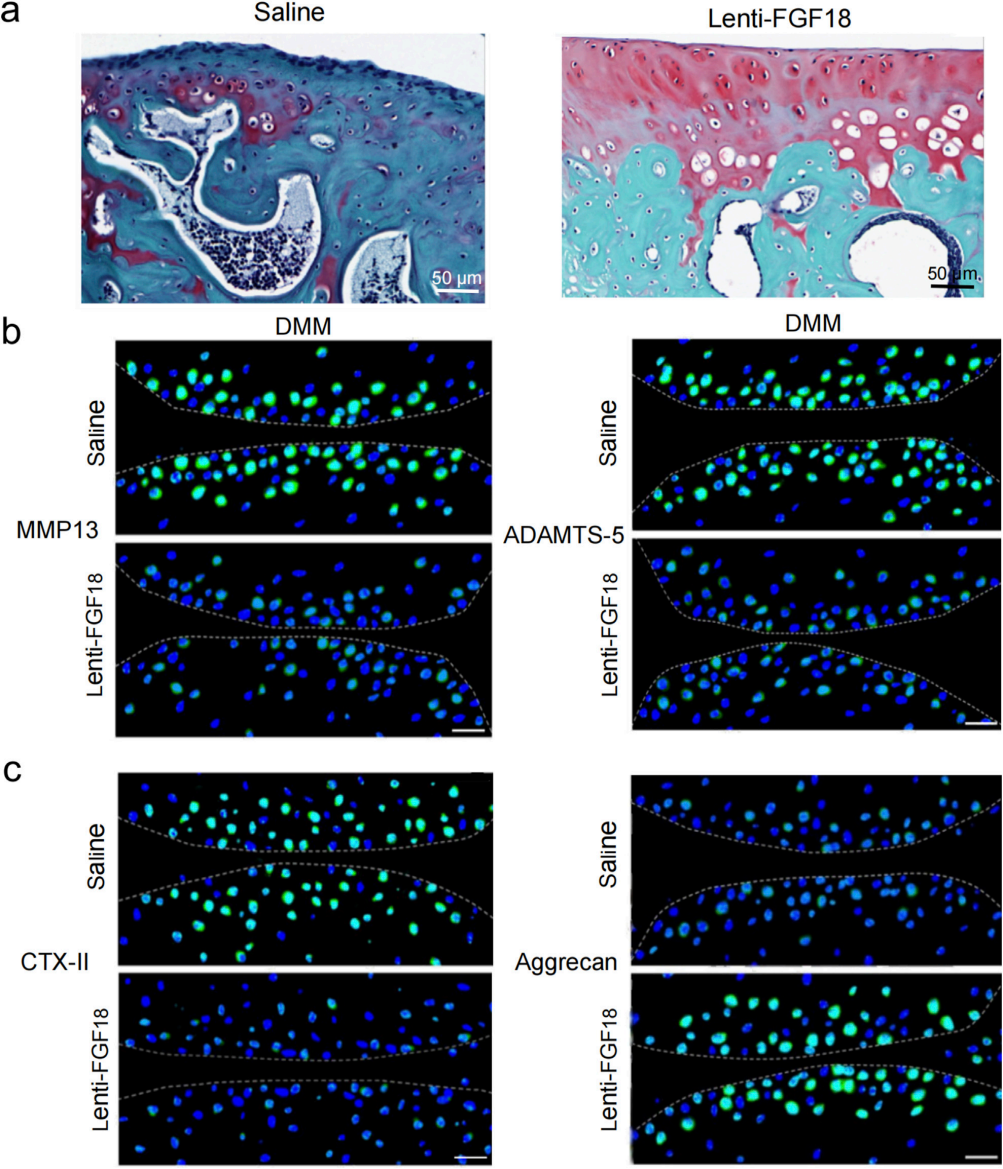
FGF18 regulates cartilage development and attenuates PTOA development. **(a)** Representative images of cartilage in DMM mice after treatment with FGF18 stained using Safranin O (n = 6 per group). Scale bar = 100 μm. **(b)** Analysis of CTX-II and Aggrecan expression in the cartilage of DMM mice after treatment with FGF18 using immunofluorescence assay. Primary antibodies: anti-CTX-II (1:500; ab3092; Abcam) and anti-Aggrecan (1:500; ab36861; Abcam), followed by FITC-conjugated secondary antibody (1:1000; Thermo Fisher Scientific). Nuclei were counterstained with DAPI. Scale bar = 50 μm (n = 6 per group). **(c)** Analysis of MMP13 and ADAMTS-5 in the cartilage of DMM mice after treatment with FGF18 using immunofluorescence assay. Primary antibodies: anti-MMP13 (1:500; ab39012; Abcam) and anti-ADAMTS-5 (1:500; ab41037; Abcam), followed by FITC-conjugated secondary antibody (1:1000; Thermo Fisher Scientific). Nuclei were counterstained with DAPI. Scale bar = 50 μm (n = 6 per group).

Next, we sought to determine whether the overexpression of FGF18 could attenuate the progression of PTOA *in vivo*. Lentiviral particles of FGF18 and saline (control) were injected into the knee joints of mice after DMM-induced PTOA. Using H&E staining and Safranin O-fast green for assessment. We observed that the overexpression of FGF18 substantially inhibited articular matrix breakdown and erosion relative to saline-treated controls (Figure 4a). Immunofluorescence analysis revealed upregulated aggrecan expression and downregulated CTX-II levels, both established biomarkers of cartilage metabolism, suggesting enhanced structural integrity of the articular matrix (Figure 4b). However, MMP13 and ADAMTS-5 were downregulated (Figure 4c), these factors break down type II collagen and aggrecan, respectively, and lead to the advancement of PTOA. From these findings, we can establish that the overexpression of FGF18 attenuated cartilage degradation under PTOA conditions.

Next, we sought to determine whether the overexpression of FGF18 could attenuate the progression of PTOA *in vivo*. Lentiviral particles of FGF18 and saline (control) were injected into the knee joints of mice after DMM-induced PTOA. Using H&E staining and Safranin O-fast green for assessment. We observed that the overexpression of FGF18 substantially inhibited articular matrix breakdown and erosion relative to saline-treated controls (Figure 4a). Immunofluorescence analysis revealed upregulated aggrecan expression and downregulated CTX-II levels (Figure 4b). Furthermore, MMP13 and ADAMTS-5 were downregulated (Figure 4c), these factors break down type II collagen and aggrecan, respectively, and lead to the advancement of PTOA.

## Discussion

The hallmark of PTOA occurrence is dependent on the balance between chondrocyte proliferation and degradation. It is marked by elevated chondrocyte catabolic activity as well as matrix breakdown and improper repair processes (Lotz and Kraus, 2010), which leads to a decrease in the number of chondrocytes and offsets the maintenance of the homeostatic balance of chondrocytes (Aigner et al., 2007; Loeser et al., 2012). Therapeutic options that are currently available for PTOA primarily focus on the reduction of pain and improvement of joint movement and not the regulation of the disease (Brown et al., 2006), therefore, determining factors that lead to the catabolism of chondrocytes and degradation of articular cartilage is an innovative and better option for PTOA treatment.

FGF18 has emerged as an important therapeutic target in osteoarthritis, with recombinant human FGF18 (sprifermin) showing promising results in clinical trials for primary OA (Hochberg et al., 2019; Eckstein et al., 2021). From our investigation, FGF18 was substantially reduced in the cartilage excised from patients with PTOA compared to the cartilage obtained from normal trauma patients without OA pathology. This establishes FGF18 as a crucial factor in the post-traumatic osteoarthrogenic mechanism. The significant downregulation of FGF18 in PTOA cartilage compared to normal tissue suggests its potential role as a key regulatory factor in PTOA development and progression, highlighting its importance as a therapeutic target specifically for trauma-induced cartilage pathology.

To further validate the role FGF18 plays in PTOA, chondrocytes from patients with PTOA were treated with pcDNA3.1-FGF18, FGF18siRNA, and their controls. The level of expression of FGF18 was upregulated in the pcDNA3.1-FGF18 group compared to its control, while its level was downregulated in the FGF18siRNA group compared to its control. The level of chondrocyte apoptosis in the group treated with FGF18 overexpression was significantly reduced while the level of proliferation was elevated compared to its control. The opposite effect was observed when FGF18 was silenced. The data presented in this report reveals that FGF18 can promote chondrogenesis progression and hinder chondrocyte death. We also performed a DMM model operation on mice and injected Lenti-FGF18 into their articular joints 3 weeks after surgical damage to the meniscus. Histological analysis of joint sections illustrated increased cartilage formation and increased articular surface thickness compared to the control group, which was injected with saline. The increase in cartilage formation was paralleled by a substantial increase in aggrecan and a decrease in ADAMTS-5, MMP13 and CTX-II. FGF18 has been shown to stimulate the proliferation of chondrocytes, increase the expression of type II collagen (Mori et al., 2014) and aggrecan, while decreasing the expression of MMP13 and ADAMTS-5 (Davidson et al., 2005). The findings reported in this study show that FGF18 has a substantial anabolic effect on chondrocytes *in vitro* and *in vivo*. The observed upregulation of aggrecan coupled with decreased CTX-II levels is particularly significant from a clinical perspective. Aggrecan serves as a validated biomarker of cartilage anabolic activity in clinical PTOA assessment, while CTX-II represents an established serum and synovial fluid biomarker for monitoring type II collagen degradation in osteoarthritis patients. This favorable shift from catabolic to anabolic cartilage metabolism suggests improved cartilage preservation that could translate to better clinical outcomes in PTOA management.

Our findings align with and extend the clinical evidence from trials investigating recombinant human FGF18 (sprifermin) in osteoarthritis patients. The FORWARD trial, a 5-year study evaluating sprifermin in knee OA, demonstrated that intra-articular sprifermin administration resulted in significant, dose-dependent increases in total femorotibial joint cartilage thickness (Hochberg et al., 2019). Specifically, patients receiving the highest dose (100 μg) exhibited sustained structural modification with effects maintained through 3–5 years post-treatment (Eckstein et al., 2021). These clinical findings corroborate our experimental observations that FGF18 promotes cartilage anabolism by inducing chondrocyte proliferation and matrix production.

However, important distinctions exist between our PTOA-focused research and the sprifermin clinical trials. First, the clinical trials primarily enrolled patients with primary, age-related OA, while our study specifically addresses post-traumatic OA, which represents a distinct pathophysiological entity. PTOA is characterized by an acute initiating event followed by rapid inflammatory responses and mechanical alterations (Anderson et al., 2011; Lotz and Kraus, 2010), whereas primary OA typically develops gradually with age-related degenerative changes. This creates a distinct therapeutic window for intervention in PTOA compared to primary OA (Thomas et al., 2017).

Second, the pathomechanisms differ substantially between these OA subtypes. In PTOA, the initial trauma triggers release of damage-associated molecular patterns (DAMPs), rapid activation of inflammatory cascades, and accelerated chondrocyte apoptosis within a concentrated timeframe (Lieberthal et al., 2015). These acute changes create a different cellular environment than the chronic, low-grade inflammation typical of primary OA. Our identification of the FGF18-FOXN2 regulatory axis represents a novel mechanism potentially specific to the trauma-induced joint damage context, which may explain why FGF18/sprifermin could be particularly effective in addressing the distinct molecular signatures of PTOA (Lieberthal et al., 2015).

Third, while sprifermin clinical trials primarily focused on cartilage thickness as the outcome measure (Hochberg et al., 2019; Eckstein et al., 2021), our study investigated multiple mechanisms of cartilage protection, including suppression of specific degradative enzymes (MMP13, ADAMTS-5) and upregulation of anabolic factors (BMP2, aggrecan). This comprehensive understanding of how FGF18 modulates both anabolic and catabolic pathways specifically in trauma-induced cartilage pathology provides stronger mechanistic insights than available from the clinical trials.

The mechanism by which FGF18 regulates chondrocyte proliferation and cartilage formation has not yet been fully established. To investigate this mechanism, an RNA-sequencing analysis was conducted to determine differentially expressed genes in PTOA chondrocytes versus normal chondrocytes from non-OA patients. FOXN2 was identified to be upregulated, implicating it as a downstream molecule for FGF18. The expression levels of FOXN2 were elevated when FGF18 was downregulated when compared to their respective controls. FOXN2 was upregulated together with MMP13 and IL-6 when FGF18 was silenced. During the degenerative development of PTOA, MMP13, a collagenase, degrades type II collagen and aggrecan in cartilage (Sophia Fox et al., 2009). The anabolism of cartilage depends on the secretion of collagen and aggrecan while the catabolism of cartilage is dependent on the expression of MMP13 and ADAMTS-5 (Verma and Dalal, 2011; Li et al., 2017). Therefore, our study surmises that FGF18 plays a pivotal role in the regeneration and protection of chondrocytes and cartilage in PTOA. This makes it a promising target for PTOA treatment.

Our findings on the FGF18-FOXN2 pathway suggest particular relevance for PTOA compared to primary OA due to the time-dependent nature of post-traumatic cartilage degeneration. Unlike primary OA, where disease onset is gradual, PTOA presents a defined therapeutic window following joint trauma (Thomas et al., 2017; Whittaker and Roos, 2019), where early intervention with FGF18 could potentially prevent the cascade of cartilage degradation events.The identification of FOXN2 as a downstream mediator of FGF18 signaling in our study offers a novel therapeutic target that may be especially relevant for intervening in the early phases following joint injury, before irreversible cartilage damage occurs. This distinction from sprifermin clinical trials, which primarily addressed established primary OA, represents a significant advancement in our understanding of how to potentially prevent PTOA development through targeted molecular interventions. While existing clinical trials focus on FGF18 for treating established OA, our findings suggest it may serve as a preventive intervention specifically in the trauma-induced OA setting, addressing an important unmet clinical need that remains distinct from primary OA management strategies.

Several limitations should be acknowledged in this study. First, our investigation used the DMM mouse model which may not fully recapitulate human PTOA complexity, and the study timeframe was limited to 8 weeks post-surgery. Second, while we identified FOXN2 as a downstream mediator of FGF18 signaling, the direct molecular mechanisms governing this interaction require further elucidation. Third, our human tissue samples were obtained from patients with established PTOA, limiting assessment of early-stage disease processes. Finally, the lentiviral delivery system used may not represent a clinically translatable approach, and optimal FGF18 delivery methods for human PTOA remain to be established. Beyond the methodological considerations outlined above, several clinical translation challenges must be addressed for FGF18 therapy in PTOA patients. Delivery optimization represents a critical hurdle, requiring development of sustained-release intra-articular formulations that maintain therapeutic concentrations while minimizing systemic exposure and potential off-target effects. Timing considerations are equally important, as the optimal therapeutic window post-injury must balance maximal chondrocyte responsiveness to FGF18 with reversible cartilage damage—too early intervention may interfere with natural healing responses, while delayed treatment may miss the critical window for preventing irreversible degradation. Additionally, patient stratification based on injury severity, joint anatomy, and individual healing responses will be essential for optimizing treatment outcomes, as our DMM model may not capture the heterogeneity of human PTOA presentations.

## Conclusion

This study establishes FGF18 as a critical regulator in PTOA pathogenesis through the newly identified FGF18-FOXN2 signaling axis. We demonstrated that FGF18 is significantly downregulated in PTOA cartilage compared to normal tissue, and its overexpression promotes chondrocyte proliferation while inhibiting apoptosis both *in vitro* and *in vivo*. Intra-articular delivery of Lenti-FGF18 in our DMM mouse model effectively prevented matrix breakdown by increasing anabolic factors (aggrecan) and suppressing catabolic enzymes (MMP13, ADAMTS-5). Our findings expand upon sprifermin clinical trial insights by highlighting PTOA-specific mechanisms and therapeutic opportunities. Unlike primary OA, PTOA presents a distinct therapeutic window following joint trauma where early FGF18 intervention could prevent irreversible cartilage damage. This time-dependent opportunity for intervention represents a crucial difference from primary OA and explains why our findings on the FGF18-FOXN2 axis have particular relevance despite ongoing clinical development of FGF18 for OA. While further research is needed to fully characterize FGF18-FOXN2 interactions and optimize delivery systems, our study provides compelling evidence that targeting this pathway represents a promising strategy for PTOA treatment, potentially addressing a significant unmet medical need in post-traumatic joint injuries. However, the therapeutic potential of the FGF18-FOXN2 axis requires further validation in larger animal models and early-phase human studies before clinical implementation can be considered.

## Data Availability

The data presented in this study are available in the Figshare repository, DOI: 10.6084/m9.figshare.30556640. The dataset consists of the processed gene expression matrix (CSV file) used for all analyses in this study.
